# Cellular and Molecular Therapeutic Targets in Inflammatory Bowel Disease—Focusing on Intestinal Barrier Function

**DOI:** 10.3390/cells8020193

**Published:** 2019-02-22

**Authors:** Ida Schoultz, Åsa V. Keita

**Affiliations:** 1 School of Medical Sciences, Örebro University, 703 62 Örebro, Sweden; ida.schoultz@oru.se; 2 Department of Clinical and Experimental Medicine, Division of Surgery, Orthopedics & Oncology, Medical Faculty, Linköping University, 581 85 Linköping, Sweden

**Keywords:** Crohn’s disease, ulcerative colitis, intestinal permeability therapeutic targets, innate and adaptive immunity

## Abstract

The human gut relies on several cellular and molecular mechanisms to allow for an intact and dynamical intestinal barrier. Normally, only small amounts of luminal content pass the mucosa, however, if the control is broken it can lead to enhanced passage, which might damage the mucosa, leading to pathological conditions, such as inflammatory bowel disease (IBD). It is well established that genetic, environmental, and immunological factors all contribute in the pathogenesis of IBD, and a disturbed intestinal barrier function has become a hallmark of the disease. Genetical studies support the involvement of intestinal barrier as several susceptibility genes for IBD encode proteins with key functions in gut barrier and homeostasis. IBD patients are associated with loss in bacterial diversity and shifts in the microbiota, with a possible link to local inflammation. Furthermore, alterations of immune cells and several neuro-immune signaling pathways in the lamina propria have been demonstrated. An inappropriate immune activation might lead to mucosal inflammation, with elevated secretion of pro-inflammatory cytokines that can affect the epithelium and promote a leakier barrier. This review will focus on the main cells and molecular mechanisms in IBD and how these can be targeted in order to improve intestinal barrier function and reduce inflammation.

## 1. Introduction 

Inflammatory bowel disease (IBD), mainly comprised of ulcerative colitis and Crohn’s disease, is characterized by symptoms such as abdominal pain, diarrhea, and weight loss. IBD affects approximately five million people worldwide and there is no curing treatment [1]. Thus, IBD patients require life-long medication and most often surgery. The exact etiology remains unknown but it is well-known that genetic, environmental and immunological factors all contribute to the disease [2]. Under normal conditions, the intestinal barrier allows only small amounts of antigens and bacteria to pass the mucosa to interact with the underlying immune cells. However, if the control of the barrier function is broken, it can lead to enhanced antigen and bacterial passage. This in turn may damage the mucosa leading to the increased production of reactive oxygen species [3] and subsequently to pathological conditions, such as IBD, and a disturbed intestinal barrier function has today become a hallmark of the disease [4].

Alterations of luminal enteric bacteria are the most important inflammation-driving environmental factor in IBD. Patients with IBD display an altered luminal gut microbiota with loss in bacterial diversity and shifts in the microbiota composition, and a possible link to local inflammation has been demonstrated [5,6,7]. Moreover, it has been suggested that the inflammatory environment in IBD favor the growth of adherent invasive bacterial strains such as *Enterobacteriaceae* and *Fusobacteria* [8]. The ability of *Fusobacterium* species to invade the intestinal epithelium also increase with the severity of IBD [9], hence indicating that these strains may play a role in IBD pathogenesis.

Except from an intestinal dysbiosis, patients with IBD have shown to have alterations in several immune cells and neuroimmune signaling pathways in the lamina propria. This might give rise to an inappropriate immune activation that can lead to mucosal inflammation, with elevated secretion of pro-inflammatory cytokines that in turn will affect the epithelial cells and promote a leaky barrier. However, it is still under debate whether the disturbed barrier is caused by a primary epithelial defect, or if it is the other way around and the increased permeability is a consequence of the inflammation. It is obvious that the human gut is complex and relies on several cellular and molecular mechanisms that allow for an intact and dynamical barrier function. The intestinal barrier consists of cellular and non-cellular components and the interaction between the epithelial cell lining and the underlying mucosal immune cells are crucial for an accurate function. This review will focus on the main cell types and molecular features involved in IBD. We will discuss cellular and molecular targets and how current and potential therapies have been developed in order to reduce inflammation and improve intestinal barrier function. 

## 2. The Intestinal Mucosa—In Health and in IBD

**Figure 1:**
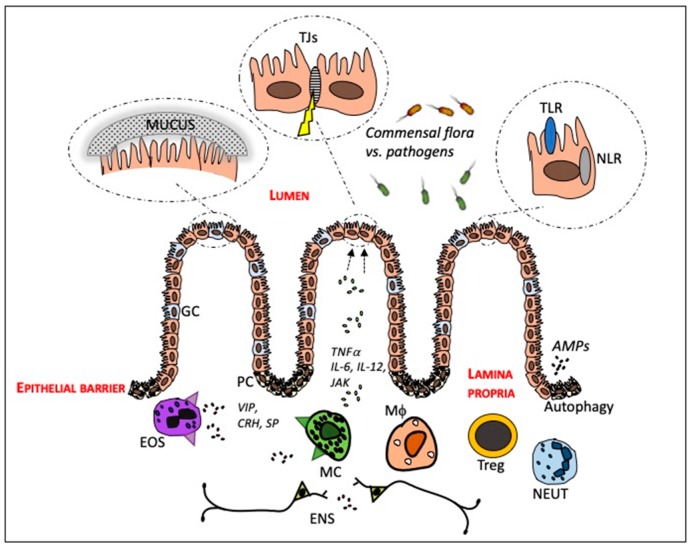
A schematic overview of the main cell types and molecular features as targets related to intestinal barrier function for therapeutic strategies in inflammatory bowel disease. AMPs = antimicrobial peptides, CRH = corticotrophin releasing hormone, ENS = enteric nervous system, EOS = eosinophil, GC = goblet cell, JAK = Janus kinases, Mϕ = macrophage MC = mast cell, NEUT = neutrophil NLR = nod-like receptor, PC = Paneth cell, SP = substance P, TJs = tight junctions, TLR = toll-like receptor, Treg = regulatory T cell, VIP = vasoactive intestinal polypeptide.

The intestinal mucosa is one of the most important barriers to the outside environment, representing the interface between the outside world and the human internal milieu. An intact barrier is maintained by the physical defense mechanism associated with the mucosal surface, the junctional complexes linking adjacent epithelial cells, and by cells of the innate and adaptive immune system. The intestinal mucosa consists of an epithelial cell lining that includes enterocytes, goblet cells and Paneth cells. In the underlying lamina propria, several immune cells that have an effect on the barrier can be found, which are in close contact with the enteric nervous system (ENS). Figure 1 illustrates an overview of the cells and molecular mechanisms that will be discussed in this paragraph.

### 2.1. The Crosstalk between the Intestinal Epithelium and Gut Microbiota 

There is a continuous interaction between the epithelial cells and the gut microbiota, which has been implicated to have a role in modulating the intestinal barrier function [10]. Animal studies indicate that the commensal microbiota is essential in shaping the intestinal barrier structure by inducing physiological paracellular permeability and fortification of the mucus layer [11]. However, a disruption of the composition of the gut microbiota will impact the host-microbial interactions and influence the intestinal physiology resulting in a diminished intestinal barrier function [10]. The first line of defense towards invading pathogens and foreign antigens is the mucus layer, a hydrated gel that covers the luminal surface of the intestinal mucosa. The mucus layer is composed of mucins secreted by the goblet cells and creates an environment that constitutes a protected habitat for the gut microbiota and particularly for specific bacterial strains that thrive in the close proximity to the epithelial cells [12,13]. Alterations of the mucus layer as well as goblet cell pathology have been associated with IBD [14]. As the mucus layer is an important habitat for the gut microbiota, a deformed mucus layer may also influence the bacterial adherence. Recently it was shown that experimental colitis in mice, induced through the exposure of dietary emulsifiers, deteriorated the protective function of the mucus layer and increased bacterial adherence and gave rise to a more pro-inflammatory microbiota [15]. 

The microbiome exists of trillions of microorganisms, mostly bacteria but also viruses, fungi and protozoa [16]. A shift in the mucosal as well as the luminal bacterial community has been associated with IBD. An increased amount of adherent invasive *E. coli* has been found in both Crohn’s disease and ulcerative colitis [7,17] and are known to affect the mechanisms that contribute to a diminished intestinal barrier function [18]. Moreover, reduced numbers of *Faecalibacterium (F.) prauznitzii* and Clostrodium clusters IV and XIVa [19,20,21,22] have been associated with IBD. This may be linked to increased pro-inflammatory metabolic properties and reduced production of short chain fatty acids (SCFA), such as butyrate, and antimicrobial peptides (AMPs) [23,24,25]. Butyrate is the main energy source for human colonocytes and is produced in the fermentation process of dietary fibers by the gut microbiota. It is essential for glucose and energy homeostasis in the intestinal epithelium [26] and has been shown to have a role in the improvement and protection of the intestinal barrier [27,28]. However, it remains unclear if the observed microbial dysbiosis in IBD is primary or merely reflects an altered microbiota-host interaction [29,30].

The symbiotic relationship between the host and gut microbiota is highly dependent on the innate immune system, where particularly the pattern-recognition receptors (PRRs) (i.e. Toll-like receptors (TLRs), Nucleotide oligomerization domain like receptors (NLRs), retinoic acid inducible gene I (RLRs), expressed in enterocytes, are an important part in the integrity of the intestinal barrier [31]. These signaling receptors are fundamental in the recognition of microbial signature molecules called pathogen/microbial-associated molecular patterns (PAMPs/MAMPs), which are expressed by most microbes [32]. Upon recognition, a direct inflammatory response against foreign microorganisms is initiated [33], where distinct groups of receptors are present in different levels to allow for integration and overlapping in the intestinal mucosa to protect the host sufficiently and keep homeostasis [34]. The intracellular NLR NOD2 has been particularly investigated in IBD as the gene that encodes the protein was the first susceptibility gene identified and is still the strongest associated risk locus for IBD [35]. NOD2 is expressed in both hematopoietic cells (e.g., lymphocytes, macrophages, dendritic cells and mast cells) and non-hematopoietic cells (e.g., enterocytes, Paneth cells, goblet cells and stem cells) [36]. In the intestine NOD2 is essential in maintaining gut barrier homeostasis through the production of AMPs by the Paneth cells [37]. 

Recognition and attachment of PAMPs/MAMPs by the innate intracellular receptors enables the identification of the foreign molecules by dendritic cells and macrophages, which act as antigen presenting cells (APCs). The dendritic cells migrate to the peripheral site of lymphoid tissue where they present antigens to T cells, which leads to the activation of several signaling pathways, and the production of pro-inflammatory cytokines, chemokines and AMPs [38] to combat the infection and protect the intestinal barrier.

A defective innate bacterial sensing has been associated with IBD and early GWAS studies have identified a risk locus in genes encoding PRRs belonging to the TLR and NLR family [35,39,40,41]. The increased susceptibility to IBD is thought to develop due to impaired pathogen recognition, which results in reduced clearance of microbes and persistent stimulation of antigens subsequently leading to elevated levels of cytokines [42]. 

One of the main cytokines associated with IBD is TNFα, a pro-inflammatory cytokine, produced mainly by activated macrophages, monocytes and T cells and is found in elevated levels both locally in the intestine and systemically in IBD patients [43,44]. The increased, persistent production of TNFα causes mucosal inflammation leading to the destruction of the intestinal barrier with increased permeability due to a reduced function of the tight junctions but also to apoptosis of intestinal epithelial cells [45,46]. The excess amount of TNFα leads to the initiation of a positive feedback loop inducing the secretion of other cytokines including IL-1, IL-6, produced mainly by mast cells, macrophages and neutrophils [47], as well as adhesion molecules, leukocytes and metalloproteinases [43,48]. The initiation of IL-6 leads to the activation of several different pathways in the adaptive immune system including Th17 and Th2 responses that exacerbate the inflammation. Subsequently this will cause negative effects on the barrier function. Another cytokine that has gained a lot of interest in IBD lately is IL-22, produced mainly by cells of the lymphoid lineage [49]. IL-22 is constitutively expressed in the small bowel and is mainly involved in the maintenance of the epithelial barrier integrity and constitutes a first line of defense towards invading pathogens. However, in the large intestine IL-22 is induced under inflammatory conditions such as IBD, initiating a signaling cascade through the JAK-STAT pathway, resulting in the induction of proliferative and anti-apoptotic pathways, as well as the production of AMPs, preventing tissue destruction and contributing to the restoration of the epithelial barrier under inflammatory conditions [49]. Moreover, it was recently shown that IL-22, IL-36γ and IL-23 are involved in a cytokine network that is induced following intestinal damage [50]. Through in vitro and in vivo experiments, it was further shown that IL-36γ signaling is a central upstream driver of the IL-23/IL-22 AMP pathway during intestinal injury. Hence, emphasizing manipulation of this cytokine pathway as a potential therapeutic target to treat intestinal damage to potentially restore a dysfunctional intestinal barrier. 

The importance of the epithelial barrier function in IBD pathogenesis has also been highlighted through GWAS studies where early studies identified a Crohn’s disease associated mutation in *DLG5*, impairing DLG5 to function as a guanylate kinase and altering the epithelial polarity [51], and polymorphisms in *OCTN1*, the organic cation/L-carnitine transporter involved in intestinal uptake [52]. Even though further studies have found contradicting results [53,54] more recent IBD candidate genes confirm the importance of intestinal barrier regulation in IBD, as reviewed by McCole et al. [55]. So far candidate genes involved in several key functions of intestinal barrier function have been identified, for example, mucus and glycoprotein regulation (*MUC19*) [56], *MUC3* [21,57,58], membrane transport (*ITLN1*) [59], epithelial differentiation (*HNF4a*) [60], stress response (*XBP1)* [61] and cell adhesion (*CDH1* [62], *LAMB1* [60,63]) including the newly identified risk gene *C1orf106* known to regulate the stability of the adherence junction [64]. Moreover, mutations in the *OSM* loci, encoding the pro-inflammatory cytokine oncostatin M (OSM), confer risk of IBD [65,66]. OSM is mainly expressed by hematopoietic cells and have been proposed to have a role in the repair of the intestinal epithelium potentially by promoting proliferation of intestinal epithelial cells [67]. Both increased levels of OSM as well as its receptor (OSMR) are found in biopsies of patients with active IBD. This phenotype has been found to be associated with anti-TNF resistant disease [65]. In addition, the genes peroxisome proliferator-activated receptor-gamma (*PPAR-γ*) [68,69] and the multidrug resistance-1 (*MDR1*) [70] are considered to be important players in gut inflammation and barrier homeostasis. 

### 2.2. Innate and Adaptive Immune Cells 

#### 2.2.1. Paneth Cells 

Paneth cells, located in the crypts of the small intestine, play an essential role in maintaining the intestinal homeostasis, particularly, by producing AMPs, which are present in high amounts in the mucus layer. It has been implicated that AMPs may have the ability to modulate both diversity and quantity of the intestinal microbiota and contribute to the clearance of invading pathogens [71], thus, protecting the intestinal epithelium towards invasion of foreign pathogens. The production and release of AMPs are dependent on autophagy. Autophagy is a cellular pathway that facilitates the degradation of cytoplasmic cargo, such as proteins, organelles but also microbial components, by delivering them to the lysosome [72]. During recent years it has become evident that autophagy is an important process of the innate immune system and affects several aspects of the mucosal immune responses essential in establishing gut barrier homeostasis [72,73]. A dysfunctional autophagy process affects the intestinal barrier function by altering the host’s ability to kill intracellular bacteria, reduce the secretion of AMPs by the Paneth cells, as well as negatively affect the mucus secretion by goblet cells [72,74]. Together the altered function of these mechanisms makes the host more vulnerable to bacterial stimuli and infectious agents as it cannot clear the bacterial products leading to increased endoplasmic reticulum stress that cannot be resolved [75], and an inability to secrete AMPs to initiate an innate immune response.

#### 2.2.2. Neutrophils 

Neutrophils are the first cells of the innate immune system reaching inflamed intestinal areas. Once they have infiltrated the intestinal epithelium they come in contact with a huge number of bacterial stimuli, get activated and then they hold the essential role of limiting microorganism invasion by recognizing and phagocytosing invading microorganisms, in order to kill them via different cytotoxic mechanisms [76]. It has become evident that neutrophils are not only involved in the acute phase of inflammation eliminating pathogens, but also are capable of modifying the overall immune response, by interacting with epithelial cells and cells of the innate and adaptive immune system such as macrophages, natural killer cells, dendritic cells, and T cells [77]. The interactions involve direct cell-cell contact or via secretion of cytokines, chemokines and chemokine receptors [76,77]. Since IBD in the colon or rectum is strongly affected by neutrophils, neutrophils are of more importance in ulcerative colitis than in Crohn’s disease [78]. In active ulcerative colitis, there is a massive infiltrate of neutrophils with a huge production of ROS and release of serine proteases, matrix metalloproteinases and myeloperoxidase leading to epithelial erosion and crypt abscesses and eventually a leakier barrier [79]. As indicated above, neutrophils are potent regulators of inflammation via the release of pro-inflammatory factors and several cytokines. However, the neutrophils have been shown to have diverse functions and the mechanisms that control the final outcome are not completely described, but these opposite functions must be tightly balanced [76]. At the early stage of mucosal inflammation in patients with IBD, neutrophils promote mucosal healing and resolution of inflammation, however, large numbers of neutrophils infiltrating in the inflamed mucosa and accumulating in the epithelia will lead to the production of inflammatory mediators which will cause an interrupted epithelial barrier [77]. 

#### 2.2.3. T Regulatory Cells 

T regulatory (Treg) cells are a subset of T cells able to suppress the activation and effector function of several immune cells involved in intestinal inflammation. Treg cells are critical for upholding immune homeostasis and for inducing and maintaining immune tolerance to luminal antigen arising from food and the commensal microbiota. Under normal conditions, the intestinal mucosa encounters numerous Treg cells, regulating lymphocytes via for example secretion of anti-inflammatory cytokines such as transforming growth factor-β and IL-10 [80]. The role of Treg cells in IBD is yet not fully elucidated, however, there is evidence that they are of importance during disease development [81] and that a Treg cell dysregulation can perpetuate the disease and the vicious cycle of inflammation [82], which subsequently might lead to an impaired barrier function. It has been shown that both Crohn’s disease and ulcerative colitis patients possess lower numbers of mucosal Treg cells during active disease [83]. In contrast, an accumulation of Treg cells has been demonstrated in active inflammatory lesions suggesting an increased migration in active phases [83,84,85]. 

#### 2.2.4. Macrophages

Intestinal macrophages represent a heterogeneous population of innate immune cells not only playing a crucial role in host defense, but also providing support to the tissue in which they reside [86]. Intestinal macrophages constantly communicate with the microenvironment and it is well known that an abnormal reaction of macrophages towards luminal bacteria and bacterial antigens can trigger and drive an exaggerated inflammatory immune reaction in the gut, which might lead to a disturbed barrier with more luminal content passing through. Macrophages are phagocytic APCs that have been described as pro-inflammatory ‘M1’ and regulatory ‘M2’ type cells, and in addition, they possess different functions depending on their localization [87]. It was recently shown that CX3CR1^+^ macrophages have the ability to rapidly respond to pathogens by migrating into the intestinal lumen in order to limit the number of bacteria breaching the epithelial barrier, thereby hindering them to cross the epithelial cells [88]. Moreover, the expression of receptors for anti-inflammatory cytokines, such as IL-10, enables the macrophages to prevent unnecessary inflammation towards harmless commensal bacteria and induce tolerance to dietary antigens [89,90]. Recently, a cross-talk between macrophages and intestinal epithelial cells was shown in a co-culture system mimicking IBD [91]. The cross-talk involved inflammatory mediators secreted from the activated macrophages causing over-expression of connexins in the epithelium. Connexins are proteins forming the gap junctions that indicates that the communication between macrophages and the intestinal epithelial cells may contribute to the dysregulation of intestinal epithelial barrier.

A marked infiltration of immature macrophages has been observed in inflamed mucosal tissues of IBD patients [92,93] resulting in large amounts of pro-inflammatory mediators, such as IL-6, TNFα, nitric oxide and reactive oxygen mediators, all known to have negative effects on intestinal barrier function [94,95]. 

#### 2.2.5. Mast Cells

Intestinal mast cells are immune cells that can be controlled by neuronal mediators. Their activation has been implicated in several types of neuro-inflammatory responses, and related disturbances of gut motility, via direct or indirect mechanisms that involve various mechanisms relevant to disease pathogenesis such as changes in epithelial barrier function or activation of immune responses [96]. Mast cells are frequently found in close proximity to nerves, and a direct interaction between nerves and mast cells often occur [97,98]. Upon neural stimulation, mast cells release a wide variety of bioactive mediators by a tightly regulated, selective secretion [99,100]. These include pre-formed mediators stored in the granules such as tryptase and histamine, and newly synthesized mediators like prostaglandins, leukotrienes, and cytokines, including TNFα, IL-3, IL-4, IL-5, IL-16 and IFNγ. Several of these mediators effect intestinal barrier function, for example tryptase, IFNγ and TNFα. Except a close connection between mast cells and enteric nerves, mast cells express receptors for neuropeptides [101,102] that together with the release of mediators demonstrate the significance of mast cells as end effector cells of the brain–gut axis in the intestinal mucosa. There is substantial evidence for the involvement of mast cells and mast cell-mediated neuroimmune interactions in IBD showing an increased secretion of mediators and an increased number and degranulation of mast cells [96,99,103], with effects on the intestinal barrier and increased permeability as a consequence. In addition, upregulated expressions of neuropeptide receptors on mast cells of both ulcerative colitis and Crohn’s disease patients have been demonstrated, with further effects on the intestinal barrier [103,104].

#### 2.2.6. Eosinophils

Eosinophils protect the host from infectious agents such as bacteria, fungi, viruses, or parasites by secretion of toxic inflammatory mediators that are stored in preformed vesicles and also synthesized *de novo* following cellular activation [105]. The major proteins secreted by eosinophils are eosinophil cationic protein (ECP), major basic protein (MBP), eosinophil derived neuroendotoxin, and eosinophil peroxidase (EPO/EPX) [106]. These proteins cause damage to tissues, and have been proposed to increase intestinal permeability, and particularly MBP [107]. During healthy conditions, the intestinal mucosa contains moderate amounts of functionally active eosinophils [105]. However, it is known that the numbers of activated eosinophils are higher in patients with active and inactive ulcerative colitis compared with controls, and interestingly, the amount of eosinophils has been shown to be higher in the inactive mucosa compared to the mucosa with an active inflammation [108]. This indicates that eosinophils may play diverse roles in the pathophysiology of IBD, that is pro-inflammatory, with negative effects on the intestinal barrier, versus tissue repair.

A close interaction between eosinophils and mast cells leading to an altered intestinal barrier function has been demonstrated. For example, during stress, substance P is released form the brain, which activates the eosinophils, leading to secretion of corticotrophin releasing hormone (CRH), which in turn activates the mast cells [109]. Upon activation, mast cells start secreting mediators that may contribute to an impaired barrier function, as described above, which further contributes to the inflammatory response. In line with this, Wallon et al. demonstrated a neuroimmune intercellular circuit from cholinergic nerves via eosinophils and mast cells in ulcerative colitis, leading to a disrupted mucosal barrier and increased permeability [104].

## 3. Current and Potential Targets for IBD Therapy 

**Figure 2:**
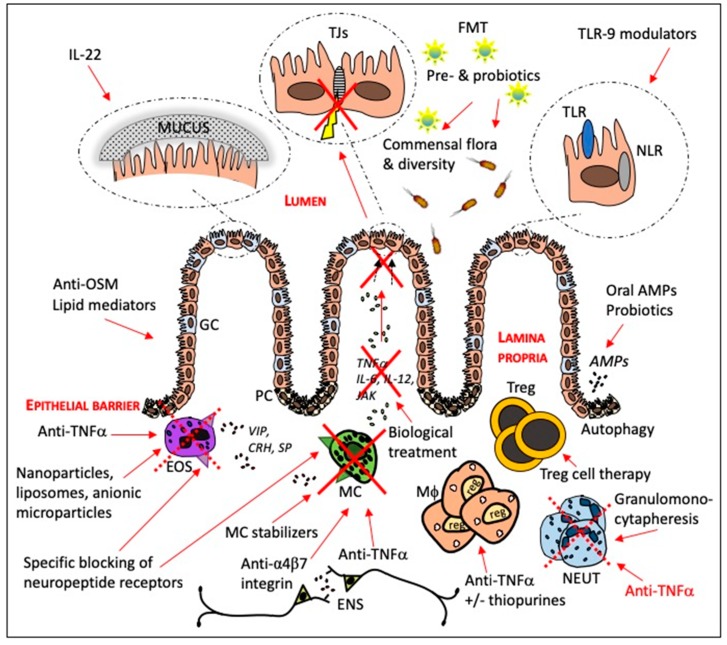
Current and potential therapies directed against targets to reduce inflammation and improve intestinal barrier function in patients with inflammatory bowel disease. AMPs = antimicrobial peptides, CRH = corticotrophin releasing hormone, ENS = enteric nervous system, EOS = eosinophil, FMT = fecal microbiota transplantation, GC = goblet cell, JAK = Janus kinases, Mϕ = macrophage MC = mast cell, NEUT = neutrophil NLR = nod-like receptor, OSM = oncostatin M, PC = Paneth cell, SP = substance P, TJs = tight junctions, TLR = toll-like receptor, Treg = regulatory T cell, VIP = vasoactive intestinal polypeptide.

There are several cellular and molecular structures as discussed above that might be targeted in the intention to find new therapeutic options for patients suffering from IBD. Figure 2 summarizes targets and therapies related to intestinal barrier function that will be discussed in this paragraph, both therapies already in use and more potential approaches.

### 3.1. Targeting Pro-Inflammatory Pathways

The microenvironment surrounding the intestinal epithelium contains cells secreting cytokines such as intraepithelial lymphocytes, dendritic cells, and eosinophils, located in close proximity to the basolateral epithelial membrane. During IBD the number and composition of these cells will change and generate a cytokine cascade that will directly affect the epithelium, resulting in a diminished intestinal barrier function. Hence, pro-inflammatory cytokines are important targets in the treatment of IBD and, by limiting the effect of these cytokines the intestinal barrier function might be restored. One strategy to indirectly target pro-inflammatory pathways and thereby restore the barrier is by targeting the proteases [110]. Proteases can be secreted both by epithelial and immune cells and can have many different functions; they may act protectively in healthy tissues, or pro-inflammatory during pathological conditions [111]. Previous studies have shown an upregulation of a large number of proteases in IBD, which for example are associated with potentiation of pro-inflammatory cytokines and degradation of tight junction proteins, leading to an increased intestinal permeability [110]. Thus, proteases might serve as efficient therapeutic targets for IBD. For example, inhibitors targeting matrix metalloproteinases, a protease mainly secreted by resident macrophages, has demonstrated good anti-inflammatory properties in mice colitis models, but less effective for this purpose in humans so far. There are many more pro-inflammatory proteases suggested as potential targets such as elastase, massively secreted by neutrophils and markedly upregulated during IBD, however, more studies are needed to define the use of protease inhibitors in IBD therapy [110].

#### 3.1.1. Antibodies against Anti-TNFα

Antibodies towards TNFα have become fundamental in the treatment of both ulcerative colitis and Crohn’s disease since the first reports of patients entering remission after treatment with the anti-TNFα antibody infliximab in 1997 [112,113]. Today several anti-TNFα agents in addition to infliximab exist on the market, i.e., adalimumab, golimumab and certolizumab for treatment of IBD [114,115]. The effect of the treatment is mainly due to the neutralization of TNFα. Upon binding to the antibody, TNFα receptor activation is prevented, resulting in reduced intestinal permeability mainly due to a reduction in apoptosis of intestinal epithelial cells as well as decreased paracellular permeability across the tight junctions [116,117]. In addition, treatment with infliximab was recently found to restore the colonic barrier to adherent-invasive *E. coli* in Crohn’s disease by blocking lipid rafts [118]. Anti-TNFα treatment also results in an increased number of Treg cells in combination with a reduced activity of inflammatory mediators and T cells [116]. More recently, high expression of OSM was found to be associated with the failure of anti-TNFα therapy [65]. These data were confirmed by animal experiments where genetic deletion or blockade of OSM in an animal model of anti-TNFα resistant intestinal inflammation significantly reduced colitis [65]. OSM is part of the IL-6 cytokine family and seems to promote intestinal inflammation and a disturbed intestinal barrier function by inducing the expression of chemokines, cytokines and adhesion factors in stromal cells of the gut that display high amounts of the OSMR-β [65,67]. Hence, OSM represents a new potential target for IBD and particular for patients not responding to anti-TNFα treatment and might facilitate restoration of the epithelial barrier function.

#### 3.1.2. Targeting the Pro-inflammatory Cytokine IL-22

Crohn’s disease patients express higher levels of IL-22 in the inflamed colon compared to ulcerative colitis patients [119]. However, IL-22 is known to have a dual role in inflammation and can have a protective role as well as in certain conditions promote inflammation [120]. Previous studies have demonstrated that treatment with recombinant cytokine or gene therapy involving IL-22 can suppress the inflammatory response and alleviate tissue injury [121]. Moreover, IL-22 has previously been found to be able to initiate the production of MUC1, a major component of the mucus layer [122,123]. Interestingly, early onset IBD patients lacking IL-10R2, a receptor of IL-22, have no expression of the abundantly glycosylated protein MUC1 [124]. Hence, IL-22 is involved in several key functions of intestinal barrier function and further research needs to be performed in order to elucidate the role of IL-22 and its potential as a therapeutic target in IBD.

#### 3.1.3. Anti-IL-6 Treatment

IL-6 has shown to be increased in serum as well as in inflamed tissue of IBD patients [125,126]. Further, IL-6 has been proposed to have an anti-apoptotic role of mucosal T cells in IBD via the induction of the anti-apoptotic genes *bcl-2* and *bcl-xl* through the activation of the STAT3 pathway [126]. A recombinant humanized monoclonal antibody (tocilizumab) of the IgG subclass directed against the soluble and membrane bound IL-6, approved for the use of rheumatic conditions, has been shown to be successful in pilot studies and case reports in IBD [127,128]. However, according to some observations tocilizumab treatment in IBD patients seems to increase the rate of intestinal perforation [129]. So far no other antibodies towards IL-6 have been assessed in clinical trials even though several have been developed and investigated in pre-clinical studies [115,130].

#### 3.1.4. Lipid Mediators as a Therapeutic Approach in IBD

A main target for many therapeutic strategies in IBD is blocking key inflammatory mediators that are triggered in the early stages of acute inflammation, such as TNFα. However, anti-TNFα treatment does not always lead to remission and, as mentioned, some IBD patients are known to be non-responders of this type of treatment [131]. Recently, resolution of the inflammatory process has been emphasized as a new therapeutic target in IBD as reviewed by Ungaro et al. [132]. This process is regulated and arranged by pro-resolving lipid mediators [133] known to reduce cellular inflammatory key events including cell proliferation, clearance of apoptotic cells, and microorganisms, hence, restoring inflamed tissue to homeostasis [134,135,136] and improved intestinal barrier function. The balance between the lipid mediators polyunsaturated fatty acids (PUFA) ω-3 and ω-6 has been recognized as particularly important in health and disease [137,138,139,140]. In ulcerative colitis the ω-6/ω-3 PUFA composition has been found to be altered compared to healthy subjects [141]. Increased levels of PUFA metabolites have been found in the mucosa of active ulcerative colitis. In addition, levels were found to correlate to disease activity [142]. Hence, indicating that ulcerative colitis patients might benefit from dietary supplementation or foods high in PUFA ω-3 [143]. However, even though some positive findings have been made regarding the use of PUFAs in the treatment of IBD the clinical studies so far are elusive and display no real evidence or support for the use of PUFAs as treatment of IBD [132].

### 3.2. Manipulation of the Intestinal Microbiota

Microbial dysbiosis has been implicated in and suggested to be predisposed to IBD [144]. The disease is associated with adherent invasive *E. coli* which correlates to gut inflammation and a perturbed intestinal barrier [145]. Restoration of intestinal microbiota dysbiosis has therefore gained interest as a treatment for IBD and studies have been performed with specific bacteria, unprocessed donor feces and parasites [144].

Probiotics are live cultured bacteria that, under specific conditions, provide health benefits to the host by influencing the composition of the gut microbiota and affect the gut immune system through their immunomodulatory properties [146]. Probiotics have been proposed to have an impact on the intestinal barrier directly, by limiting the colonization of pathogenic bacteria as well as indirectly by interacting with innate immune cells, leading to the production of IL-22 and the initiation of specific mucin genes [147]. Several studies have been performed to evaluate probiotics in the treatment of IBD [148]. A modest effect of *E. coli* Nissle 1917 has been demonstrated in ulcerative colitis patients with mild disease [149]. Previous findings indicate that the effect might be partially due to activating AMP production [150,151]. In addition, experiments in mouse models show that colonization with *E. coli* Nissle 1917 upregulates the mRNA and protein expression of the tight junction protein ZO-1 and when given orally was able to reduce experimental colitis and improve intestinal barrier function [152]. However, current knowledge indicates that probiotics have limited clinical effects in patients suffering from Crohn’s disease [148]. Even though the evidence for use of probiotics in IBD remain sparse, next generation probiotics that take into account the difficulties in culturing anaerobic cultures as well as the lack of specific effects may open up for new possibilities of probiotic treatment in IBD [153].

Prebiotics, dietary fibers, (i.e. non-digestible polysaccharides) are fermented by the gut microbiota and in that process SCFA are generated, of which butyrate is the most abundant and known to have anti-inflammatory properties as well as potential barrier promoting effects [27,28]. Previously, it has been shown that the supernatant of *F. prausnitzii*, one of the main butyrate producers [154], enhances the intestinal barrier function in a mice model of colitis by affecting paracellular permeability [155]. Hence, indicating a potential role for *F. prausnitzii* and prebiotics in the treatment of IBD. Moreover, low levels of *F. prausnitzii* predicted relapse after treatment with infliximab discontinuation [156]. Recently, it was shown that the dietary fiber yeast beta-glucan was able to reduce mast cell-induced intestinal permeability across the mucosa of Crohn’s disease patients and control subjects [157], suggesting, that prebiotics do not only act via induction of colonization of the gut microbiota but also elicit direct effects on the intestinal barrier. In addition, a whey protein component, casein glycomacropeptide was found to have a similar effect as 5-ASA in a small pilot study enrolling ulcerative colitis patients [158], and likewise, the formulation phosphatidylcholine LT-02 showed beneficial effects [159]. Currently dietary supplements containing these substances are being developed for further investigation.

Fecal microbiota transplantation (FMT), the transfer of unprocessed donor feces in the colon through either enema, colonoscopy, or a naso-jejunal feeding tube is a well-established treatment of recurring *Clostridium difficile* infection [160]. The treatment has during the last decade gained a lot of interest in IBD and clinical trials have been performed in both ulcerative colitis and Crohn’s disease patients. In ulcerative colitis, some clinical trials have been reported to have modest positive effects of FMT compared to placebo [161,162,163]. However, the variation in the outcome might be due to donor characteristics, abundance of bacterial species, as well as how compatible the recipients are to the donors [164]. In Crohn’s disease the evidence for FMT is scarce and only a few case reports have been published [165,166]. However, FMT is still of interest in IBD and may elicit a beneficial effect through various routes. Recently, a case series of FMT using sterile-filtered fecal water was effective in the treatment of *Clostridium difficile* infections [167]. Thus, indicating that colonization is not essential in order to achieve a positive effect, instead other factors such as bacterial components and metabolites might be important for a successful outcome [167]. Even though the focus in FMT is on the bacterial content, the intestinal virome expressed as bacteriophages might be significantly different in IBD and represent a potential clinical target [168].

Moreover, the disturbance in the secretion of AMPs associated with IBD, have opened up for the oral administration of defensins as a promising therapeutic option [169]. By utilizing specific modifications the peptides could be enriched in the mucus at different locations of the intestine and protect the epithelial layer from close contact and attack from bacteria residing in the lumen [169]. Recent findings presented at the 2018 congress of the European Crohn’s and Colitis organization (ECCO) indicate that oral delivery of human β-defensin 2 induces an increased diversity of the gut microbiota and is effective in the treatment of experimental colitis in mice [170]. The development of new therapeutic molecules targeting Crohn’s disease is currently on-going [171], even though the clinical use is still at an early stage.

### 3.3. Neutrophils as Targets

Targeting neutrophils and their inflammatory mediators with negative effects on the intestinal barrier is an opportunity that should be explored to identify new effective IBD therapies. As mentioned above, neutrophil infiltrates more often occur in colon and rectum, subsequently, treatment to target neutrophils is in general more efficient in patients with ulcerative colitis compared to Crohn’s disease [78,172]. There are several key neutrophil related proteins with links to ulcerative colitis that are potential therapeutic targets, and for example, neutrophil related proteins like CXCR1, CXCR2 and matrix metalloproteinase 9 have entered clinical development [79]. Another way to target neutrophils is by adsorptive granulomonocytapheresis where the neutrophils are phagocytosed by CD19 B cells to become regulatory B cells that produce the anti-inflammatory cytokine interleukin-10 [173], which have strengthening effects on the intestinal barrier. The efficacy outcomes of this treatment have been impressive as well as disappointing and only patients without deep ulcers or extensive loss of mucosal tissue have responded well to granulomonocytapheresis and achieved a favorable long-term disease course [173]. More clinical settings are needed to fully evaluate the efficacy of neutrophil-targeted therapy in IBD. Furthermore, it is important that the therapy only modulates neutrophil activity and not completely silence it, thereby abolishing the destructive inflammation and tissue damage without compromising host-defense.

As for macrophages, one way to target neutrophils and their negative effect on barrier function is by blocking pro-inflammatory cytokines. Recently, Zhang et al. [174] showed that anti-TNFα therapy significantly downregulated the infiltration of neutrophils in inflamed mucosa of Crohn’s disease and ulcerative colitis patients. Notably, anti-TNFα antibodies could inhibit neutrophils to produce pro-inflammatory mediators, such as ROS, calprotectin, IL-8, IL-6, and TNFα. These results indicate that the inhibition of TNFα modulates intestinal homeostasis through balancing the immune responses of neutrophils, which also might lead to an improved barrier function.

### 3.4. The Use of Treg Cell Therapy

Due to the potent suppressive mechanisms of Treg cells, they should represent a promising therapeutic strategy for patients with IBD. If the dysregulation of Treg cells, as observed in some IBD patients, could be inhibited it would lead to a decreased inflammation and consequently also an improvement of the intestinal barrier function. But even though the use of Treg cells as a therapy has plenty advantages, there are many questions that must be answered before Treg cell therapy can be considered in the context of IBD. The most important concerns are related to (1) the efficacy during an ongoing inflammation (2) how to correctly traffic the infusion of cells (3) risk of Treg cells to convert into effector cells leading to disease worsening, and (4) the significant influence of the microbiota on the outcome of the treatment [175,176]. So far, there is, to our knowledge, only one study published testing the efficacy of Treg cell therapy in IBD [177]. In this phase I/IIa trial, cloned ovalbumin-specific Treg cells were administrated intravenously to 20 patients with refractory Crohn’s disease. Results showed that the administration of Treg cells was well tolerated and had dose-related efficacy and the ovalbumin-specific immune response correlated with clinical response, supporting immune-suppressive mechanisms of ovalbumin-specific Treg cells. However, this immune therapy approach warrants further clinical and mechanistic studies. So far, no additional clinical studies to treat IBD and thereby enhance the intestinal barrier function with Treg cell therapy have been published, even though there is hope that it will soon be deployed in the setting of IBD, and prove more effective than the current nonspecific immunosuppressive therapies. In addition, the questions listed above need to be answered in experimental models of IBD while translational strategies are developed.

### 3.5. Macrophages as Therapeutic Targets

One of the major therapeutic objectives in the management of IBD is mucosal healing of the intestine. As mentioned, a key role in this process is played by regulatory macrophages [178]. It has been shown that anti-TNFα antibodies can induce regulatory macrophages in IBD patients, which promote wound repair [179,180], leading to less inflammation and improved barrier function. Vos et al. showed that anti-TNFα in combination with thiopurines enhanced the induction of regulatory macrophages both in number and in immunosuppressive potential compared to anti-TNFα monotherapy [179]. Unfortunately, a relatively large proportion of IBD patients are intolerant to thiopurines and in this group, anti-TNFα/thiopurine combination therapy is not possible [181]. Therefore, ongoing research aims to find alternatives for combination therapy with anti-TNFα. Nuclear Enriched Abundant Transcript 1 (NEAT1) is a novel nuclear long non-coding RNA which localizes in specific nuclear structures and is involved in the immune response in a variety of ways [182]. Recently, Liu et al. [182] showed that inhibition of the NEAT1 suppressed the inflammatory response by modulating the intestinal epithelial barrier and through exosome-mediated polarization of macrophages in IBD. These results might reveal a potential strategy in IBD therapy by targeting NEAT1 to improve barrier function and thereby dampen the ongoing inflammation, but more studies are needed.

### 3.6. Mast Cells as Therapeutic Targets

As mentioned, mast cells are important players in mucosal immune responses and in the regulation of intestinal barrier function in IBD. The elevation of mast cell numbers in IBD promotes them as potential therapeutic targets using pharmacological agents against numerous biologically active molecules secreted by them. For example, one approach to prevent pathological mast cell activation and thereby improving mucosal barrier function has been the use of mast cell stabilizers such as ketotifen, tranilast, histamine H1-receptor antagonists, serotonin 5-HT3 receptor antagonist and disodium cromoglicate [183,184,185,186]. The exact mechanisms of action are yet not clear and the efficiency of mast cell stabilizers, and for example histamine H1-receptor antagonist in the treatment of gastrointestinal disorder, is so far uncertain. Studies have shown promising effects of mast cell stabilizing agents in irritable bowel syndrome [187,188], however, there are very few studies on IBD patients [184,189]. The first study to provide evidence for a potential role of ketotifen in treatment of IBD is a case report by Marshall et al. [183] in 1998. Ketotifen was given to IBD patients with active colitis who were intolerant to 5-ASA. The treatment resulted in improved symptoms with less symptomatic burden and decreased stool frequency. However, the study is uncontrolled and only includes three patients, but nevertheless it highlights the potential role of mast cell stabilizers as a therapeutic tool in colonic inflammation.

Vedolizumab is a drug recently introduced in the management of IBD. It is a monoclonal antibody that binds to α4β7 integrin resulting in gut-selective anti-inflammatory activity by blocking lymphocyte trafficking to gut mucosa [190,191]. It has been shown that α4β7 integrin is critical not only for lymphocyte homing but also for homing of mast cells [192], which could help to explain the positive response of IBD patients to the therapy with Vedolizumab [191,192]. Except from therapy using mast cell stabilizers there are anti-inflammatory drugs that have shown to reduce mast cell infiltration, and thereby consequently also reduce the negative effects on the intestinal barrier caused by mast cells. For example, oral treatment with Mesalamine (mesalazine), a 5-ASA compound that is the first-line treatment for patients with mild-to-moderate ulcerative colitis reduced mast cell infiltration in patients with irritable bowel syndrome [193] and might have the same working mechanism also during ulcerative colitis.

It has been demonstrated that barrier dysfunction caused by chronic stress in rats, or by exposure of human biopsies to stressors, was inhibited by blocking the receptors for CRH, substance P and vasoactive intestinal polypeptide [101,102,194]. A novel approach could therefore be to target surface receptors known to be involved in mast cell degranulation in order to achieve a more directed pharmacological therapy for patients with IBD.

### 3.7. Eosinophils as Therapeutic Targets

Blocking of eosinophils, and thereby inhibiting the secretion of mediators may be a potential biological therapy to target the improvement of the intestinal barrier. However, given the diverse roles that eosinophils have, both being pro-inflammatory and repairing, it would be critical to accurately identify the mechanism for each process to be able to reach a balance of inhibiting the inflammatory effects without interfering with the repair mechanism. Although this has only been explored in experimental models and it remains to see if blockers will find practical use also in the treatment of IBD patients [195]. As for mast cells, anti-TNFα most likely has an effect also on the eosinophils. In a case report study by Turner et al. [196], eight children with refractory eosinophilic enterocolitis were treated with infliximab. Treatment resulted in rapid and complete clinical remission in 75% of the children. Even if this is an uncontrolled report and further studies are needed, it indicates an effect of anti-TNα antibodies on eosinophils.

Carlson et al. [197] showed the mucosal release of ECP and EPO/EPX was 10-20 times increased in patients with ulcerative colitis as compared with healthy controls. In a commentary by Al-Haddad et al. [195] it is speculated that this pathophysiological difference provides opportunities for new therapeutic interventions in ulcerative colitis and Crohn’s disease by the potential use of anionic microparticles, nanoparticles, or liposomes. These can all bind to the positively charged eosinophilic proteins in the gut mucosa of IBD patients, hindering them to exert their negative effects on intestinal permeability and thereby restore the barrier function. However, the negatively charged mucus layer in the small intestine and colon must be considered in the design of the therapy using electrostatically charged delivery systems.

## 4. Conclusions

The intestinal barrier is a complex structure and crucial for its function are interactions between the epithelial cell lining and the underlying mucosal immune cells. Alterations in these interactions might give rise to pathophysiological conditions such as IBD. There are several cellular and molecular structures as discussed above that might be targeted in the intention to improve intestinal barrier function, and find new therapeutic options for patients suffering from IBD. However, future research is essential in order to translate the knowledge from pre-clinical studies and early clinical findings to use in a daily clinical setting. Particularly, the mapping of the gut microbiota composition in relation to genetics in IBD will allow for a more personalized medical approach.
